# The Role of Adenosine in Overcoming Resistance in Sarcomas

**DOI:** 10.3390/ijms252212209

**Published:** 2024-11-14

**Authors:** Marlid Cruz-Ramos, Sara Aileen Cabrera-Nieto, Mario Murguia-Perez, Fernanda Sarahí Fajardo-Espinoza

**Affiliations:** 1 Investigadora por México del Consejo Nacional de Humanidades, Ciencias y Tecnologías (CONAHCYT), Mexico City 03940, Mexico; 2 Facultad de Ciencias de la Salud, Universidad Anáhuac México, Huixquilucan 52786, Mexico; sara.cabrera32@anahuac.mx (S.A.C.-N.); fernanda.fajardoe@anahuac.mx (F.S.F.-E.); 3 Laboratorio de Anatomía Patológica e Inmunohistoquímica Especializada DIME, Hospital Médica Campestre, León 37180, Mexico; drmariopatologia@gmail.com; 4 Departamento de Patología Quirúrgica, UMAE Hospital de Especialidades No. 1, Centro Médico Nacional Bajío, Instituto Mexicano del Seguro Social, León 37328, Mexico

**Keywords:** sarcoma, adenosine, chemotherapy resistance, immunotherapy

## Abstract

Resistance to systemic therapies in sarcomas poses a significant challenge to improving clinical outcomes. Recent research has concentrated on the tumor microenvironment’s role in sarcoma progression and treatment resistance. This microenvironment comprises a variety of cell types and signaling molecules that influence tumor behavior, including proliferation, metastasis, and resistance to therapy. Adenosine, abundant in the tumor microenvironment, has been implicated in promoting immunosuppression and chemoresistance. Targeting adenosine receptors and associated pathways offers a novel approach to enhancing immune responses against tumors, potentially improving immunotherapy outcomes in cancers, including sarcomas. Manipulating adenosine signaling also shows promise in overcoming chemotherapy resistance in these tumors. Clinical trials investigating adenosine receptor antagonists in sarcomas have fueled interest in this pathway for sarcoma treatment. Ultimately, a comprehensive understanding of the tumor and vascular microenvironments, as well as the adenosine pathway, may open new avenues for improving treatment outcomes and overcoming resistance in sarcoma. Further studies and clinical trials are crucial to validate these findings and optimize therapeutic strategies, particularly for osteosarcoma. This study provides a literature review exploring the potential role of the adenosine pathway in sarcomas.

## 1. Introduction

Sarcomas represent a diverse group of approximately 80 entities classified by the World Health Organization (WHO) as soft tissue sarcomas based on their morphological, immunohistochemical, and molecular characteristics. Clinical presentation is also essential for determining prognosis and guiding treatment decisions. Multidisciplinary management is vital for improving clinical outcomes [1].

Standard systemic therapies for sarcomas include doxorubicin (DOX), cisplatin (CDDP), ifosfamide (IFO), methotrexate (MTX), gemcitabine (GEM), docetaxel (DTX), and trabectedin. These drugs are effective in treating both soft tissue sarcomas and osteosarcoma (OS). For localized and metastatic soft tissue sarcomas, DOX and IFO are considered first-line options [2]. OS, the most common bone tumor, is typically treated with the MAP regimen, which combines CDDP, DOX, and high-dose MTX [3]. Despite advances in systemic therapies, patient survival outcomes remain limited, with chemotherapy resistance likely contributing to suboptimal clinical results [4,5].

Cellular resistance mechanisms in sarcomas are well characterized, and ongoing research continues to explore strategies to modulate these mechanisms and enhance treatment efficacy. However, in recent years, the tumor microenvironment (TME) has emerged as a primary focus in understanding resistance mechanisms in sarcomas [6].

Tumor research has increasingly focused on the tumor microenvironment (TME), which promotes tumor cell proliferation, metastasis, anti-apoptotic mechanisms, and drug resistance [7]. For instance, osteosarcoma (OS) exists within the bone microenvironment, a complex niche containing bone, stromal cells, blood vessels, immune cells, and a mineralized extracellular matrix [8]. This environment enables signaling interactions between OS and the surrounding bone, inducing cytokines, chemokines, and growth factors. Targeting cellular components within the bone microenvironment can suppress OS growth and potentially improve patient survival [9].

Mifamurtide was one of the first drugs to demonstrate the potential of modulating the tumor microenvironment (TME) in osteosarcoma (OS). This molecule activates monocytes and macrophages, which then produce cytokines such as tumor necrosis factor and interleukins-1, 6, 8, and 12, along with adhesion molecules like lymphocyte function-associated antigen-1. This activation promotes the shift from M2 (anti-inflammatory) macrophages to M1 (pro-inflammatory) macrophages [10,11]. The results from the multicenter INT-0133 trial indicate that patients treated with mifamurtide had a 6-year overall survival rate of 78%, compared to 70% for those who did not receive the drug [12]. In some European countries, mifamurtide has been approved to prevent lung metastasis in patients with localized OS [11,13].

The vascular microenvironment has been extensively studied in sarcomas and osteosarcoma (OS), revealing that the expression of vascular endothelial growth factor (VEGF) and its receptor VEGFR-2 correlates with poor outcomes in OS patients. Tyrosine kinase inhibitors (TKIs) have emerged as a targeted approach for this pathway. In a metastatic OS setting, regorafenib, a multi-target TKI, has been shown to reduce disease progression and improve survival by 3.5 months compared to 1.7 months for placebo [14,15]. Similar results have been observed with other TKIs, including sorafenib [16], pazopanib [17], apatinib [18], and cabozantinib [19]. Despite these advances, overall response rates and survival outcomes with these drugs in OS remain limited, prompting clinical trials to test combinations of chemotherapy and targeted therapies to enhance effectiveness. Antiangiogenic therapy has also shown efficacy in other sarcoma subtypes, such as extraskeletal myxoid chondrosarcoma [20,21], solitary fibrous tumors [22], desmoid tumors [23,24,25], and alveolar soft part sarcoma [26,27,28,29,30].

Identifying pathways that contribute to chemoresistance, modulate immune response, and influence angiogenesis within the tumor microenvironment (TME) could significantly improve clinical outcomes in sarcomas, including osteosarcoma (OS) [31,32]. This work aims to explore the adenosine (ADO) pathways and their roles in conferring chemoresistance to standard sarcoma therapies, as well as their potential in enhancing outcomes in soft-tissue and bone sarcomas. We examine the effects of the canonical ADO pathway on treatment response, shedding light on a critical aspect of cancer management. Additionally, we investigate how the ADO pathway impacts the inflammatory response, which may contribute to the limited success of immunotherapy in sarcomas. Finally, we provide evidence supporting the ADO pathway’s potential in optimizing sarcoma treatment strategies.

## 2. Methods

This work is a narrative literature review focusing on the role of the adenosine pathway in sarcomas and its relationship with chemotherapy resistance, immunotherapy, and innovative therapeutic strategies. The literature search was conducted using PubMed, Scopus, Web of Science, and Google Scholar, covering articles published from January 2000 to July 2024. Key search terms included “adenosine”, “adenosine pathway”, “sarcoma”, “chemotherapy resistance”, “immunotherapy”, “tumor microenvironment”, “nanomedicine”, “targeted therapies”, and “inflammatory markers”. Relevant articles from peer-reviewed journals were included, without language restrictions, while conference abstracts and studies not directly related to the review objectives were excluded.

## 3. Adenosine Pathway

The adenosine (ADO) pathway, a critical factor in numerous diseases, holds special significance in cancer research. This review focuses on both the canonical and non-canonical ADO pathways [33,34], with particular emphasis on the canonical pathway, which has been extensively studied in cancer.

### 3.1. Canonical Adenosine Pathway

Adenosine is a purine nucleoside essential for various bodily systems, including the nervous, reproductive, vascular, cardiac, renal, respiratory, hepatic, and immune systems [35]. It is produced from the metabolism of adenosine triphosphate (ATP) or nicotinamide adenine dinucleotide (NAD+) and is transported by the nucleoside transporter ENT1 and other transporters.

ATP is converted to 5′-adenosine monophosphate (AMP) by ectonucleoside triphosphate diphosphohydrolase 1 (*ENTPD1*, also known as CD39) and then is fully converted into adenosine by ecto-5′-nucleotidase (*NT5E*, also known as CD73). Once produced, ADO is released and acts through four receptors coupled to G proteins called A1 (A1R), A2A (A2AR), A2B (A2BR), and A3 (A3AR) receptors [33,36]. The activation of these adenosine pathways is crucial for various physiological functions.

Adenosine receptors (ARs) modulate cellular responses by either inhibiting or stimulating adenylate cyclase, thereby increasing or decreasing intracellular cyclic adenosine monophosphate (cAMP) levels. These receptors can also activate other signaling pathways, such as the mitogen-activated protein kinase (MAPK) pathway [37,38]. Activation of these pathways requires minimal extracellular adenosine (ADO) concentrations; however, stress conditions like hypoxia, cancer, tissue damage, or inflammation can significantly increase ADO levels [33,39]. Extracellular ADO is generated by a balance between its production and inactivation, which, in turn, influences ATP signaling pathways. Both extracellular ADO dynamics and intracellular metabolism, alongside compartmentalization of the ADO system, play vital roles in numerous biological processes, including cancer [40].

Endogenous ATP can be released in substantial amounts during necrosis, apoptosis, and cellular damage, as well as in response to non-injury-related stimuli, such as vesicle exocytosis or ion channel activation via nucleotide transporters and connexin/pannexin hemichannels [40,41]. Extracellular ATP (eATP), for example, serves as a “damage sensor”, attracting phagocytic cells to inflammation sites and alerting the immune system to tissue damage or infection. This eATP is essential for triggering inflammation and releasing pro-inflammatory interleukins like interleukin-1β. These effects are primarily mediated by purinergic receptors (P2X and P2Y), with P2X7, a ligand-gated ion channel, being particularly involved in immunomodulation and tumor progression [41,42].

The adenosine (ADO) pathway is essential in maintaining optimal energy metabolism, which is vital for health. Disruptions in this energy balance can lead to various health disorders. AMP-activated protein kinase (AMPK) is a key fuel-sensing enzyme involved in regulating ATP production and expenditure [43,44]. AMPK serves as a highly conserved sensor of intracellular adenosine nucleotide levels and is activated by ATP depletion alongside increases in AMP or ADP. When activated, AMPK initiates downstream pathways, including the phosphorylation of Raptor at S792, which is crucial for inhibiting mTOR during energy stress or low energy states (high AMP/ATP ratio) [43,45].

Under energy stress conditions, AMPK is activated through liver kinase B1 (LKB1), TGF-β-activated kinase 1 (TAK1), and calcium/calmodulin-dependent protein kinase kinase β (CaMKKβ) [43,46]. The LKB1/AMPK pathway can act as a tumor suppressor, inhibiting tumor cell growth [47].

However, AMPK can also act as a tumor promoter by allowing tumor cells to adapt metabolically. This adaptation enables tumor cells to withstand stress conditions and eventually develop chemoresistance, a topic that will be discussed in further detail later.

### 3.2. Canonical Adenosine Pathway in Sarcomas

To explore the potential role of the adenosine (ADO) pathway in sarcomas, we examined the presence of key pathway components in these tumors. Studies have indicated the presence of CD39 and CD73 in sarcoma cells and in patient samples, suggesting an active ADO pathway in these tumors [48,49]. In a 2015 study, Dr. Hayes and colleagues used mass spectrometry and immunohistochemistry (IHC) to demonstrate CD39 expression in sarcomas such as liposarcoma, fibrosarcoma, dermatofibrosarcoma, and leiomyosarcoma. Notably, adjacent stromal tissue cells did not express CD39. Additionally, the researchers employed a patient-derived xenograft (PDX) model of recurrent fibrosarcoma expressing CD39 in a murine model deficient in T, B, and natural killer (NK) cells. They found that inhibiting CD39 with a specific inhibitor reduced tumor size and prolonged animal survival [49].

Another research group investigated the potential role of CD73 in rhabdomyosarcoma, specifically in fusion-negative rhabdomyosarcoma (FN-RMS), which accounts for over 80% of all rhabdomyosarcoma cases. The mechanisms driving FN-RMS pathogenesis are not well understood, but CD73 was identified as a TWIST2-regulated gene involved in FN-RMS development. Experimental knockdown of CD73 by this group led to reduced pathogenic growth of FN-RMS both in vitro and in vivo. Additionally, CD73 knockdown caused cell cycle arrest, decreased cell migration, and initiated the myogenic program in FN-RMS cells. The study authors concluded that CD73’s role in FN-RMS is linked to its enzymatic activity and activation of the purinergic signaling pathway [50].

Dr. Aoki’s group also investigated CD73 expression in epithelial sarcoma tissue and discovered that CD73 forms a complex with emmprin, a protein linked to the production of matrix metalloproteinase type 2 (MMP-2). This complex forms between fibroblasts and tumor cells. When fibroblasts were co-cultured with sarcoma cells, CD73 knockdown via small interfering RNA (siRNA) suppressed MMP-2 production. Notably, emmprin levels were higher in tumor cells than in fibroblasts, whereas CD73 was present in both cell types. The use of CD73-specific inhibitors did not reduce MMP-2 production, but CD73 neutralizing antibodies did lead to decreased MMP-2 levels, suggesting that CD73 influences MMP-2 production through a non-enzymatic mechanism. This study supports the presence of CD73 in sarcoma cells and highlights its role in the tumor microenvironment, potentially promoting tumor invasion [51].

Research has also indicated that AMPK activation may play a role in sarcomas. Dr. Morishita and colleagues explored the therapeutic potential of promoting mitochondrial proliferation in human osteosarcoma (OS) cells through the activation of peroxisome proliferator-activated receptor-gamma coactivator-1 alpha (PGC-1α) by AMPK. They used aminoimidazole carboxamide ribonucleotide (AICAR), a nucleoside analog initially developed as a cardioprotective agent, to activate the AMPK/PGC-1α/TFAM pathway for mitochondrial biogenesis, thereby targeting cancer cell energy metabolism. AICAR is phosphorylated to AICA-ribotide (ZMP), which mimics AMP and activates AMPK. The study demonstrated that AICAR induced mitochondrial apoptosis in human OS by increasing PGC-1α expression via AMPK activation, leading to mitochondrial proliferation. This increased apoptotic activity and mitochondrial proliferation in AICAR-treated OS cells inhibited OS cell growth. These findings suggest that reduced mitochondrial numbers contribute to the progression of human OS, and that enhancing mitochondrial biogenesis with AICAR through AMPK activation exerts anticancer effects on human OS by promoting mitochondrial proliferation and apoptosis [52].

An alternative agent, 6-gingerol, has been found to activate the AMPK signaling pathway in osteosarcoma (OS). Research suggests that 6-gingerol inhibits OS cell growth and reduces cell viability in a dose-dependent manner. Furthermore, 6-gingerol significantly increases the number of cells arrested in the sub-G1 phase of the cell cycle and activates caspase cascades, affecting the cellular levels of Bcl2 and Bax through AMPK activation. This study provides additional evidence for the potential role of AMPK in OS [53].

In non-pathological bone, the A2BR receptor is activated when adenosine (ADO) concentrations reach the micromolar range. Bone injury increases extracellular ATP (eATP), which is then metabolized to ADO by cell surface ectonucleotidases. Stress conditions, such as inflammation and oxidative stress, upregulate A2BR expression, promoting mesenchymal stem cell differentiation into osteoblasts and supporting bone formation in vivo [54]. ATP release channels also play a role in osteosarcoma (OS). Connexin43 (Cx43), a gap junction protein facilitating intercellular communication, suppresses the proliferation of human OS U2OS cells by inhibiting the G1-to-G2 cell cycle transition. This effect is attributed to the accumulation of hypophosphorylated retinoblastoma protein, which decreases the kinase activities of cyclin-dependent kinases (CDKs) 2 and 4. Cx43 appears to inhibit U2OS cell proliferation by increasing p27 protein levels through post-transcriptional regulation [55,56]. Cx43 regulation involves small ubiquitin-like modifiers (SUMOs) and the SUMO-conjugating enzyme UBc9, which is overexpressed in OS. Silencing UBc9 with siRNA has been shown to inhibit OS cell proliferation and migration while increasing sensitivity to thymidine kinase/ganciclovir (HSC-TK/GCV), suggesting that Cx43 expression may enhance sensitivity to conventional chemotherapy [56]. ALMB-0168 is a humanized monoclonal antibody that specifically binds to the extracellular domain of Connexin43 (Cx43). It activates Cx43 hemichannels in osteocytes, enhancing ATP release in cultured osteocytes and osteocytes in murine models [57]. In wild-type (WT) Cx43 murine models, ALMB-0168 has been shown to reduce bone cancer growth, although this effect is not observed in Cx43 knockout mice. Additionally, ALMB-0168 increases cytotoxic lymphocyte levels (CD3/CD8+ T cells) and helper T-cell levels (CD3/CD4+), improves survival rates, and reduces tumor metastasis [57,58]. The P2X7 receptor is highly expressed in osteosarcoma (OS) tissues and supports the growth and spread of human HOS/MNNG cells through PI3K/AKT/GSK3β and mTOR/HIF/VEGF signaling pathways. Interestingly, extracellular ATP (eATP) can increase plasma membrane permeability by opening P2X7 receptor (P2X7R) pores, facilitating the entry of cytotoxic agents like doxorubicin (DOX) [59]. Both P2X7RA and P2X7RB receptor isoforms are present in OS tissue, with P2X7RB-positive tumors exhibiting higher cell density depending on the tumor microenvironment (TME) [60]. Shock wave-induced ATP release from OS U2OS cells can also enhance methotrexate (MTX) uptake and cytotoxicity by increasing cell membrane permeability in a P2X7 receptor-dependent manner [61].

Adenosine and its receptors, extracellular ATP (eATP), and transport channels play a potential role in osteosarcoma (OS) carcinogenesis pathways, positioning them as promising targets for disease management and for reversing resistance mechanisms.

## 4. Canonical Adenosine Pathway and Chemotherapy Resistance

### 4.1. Cisplatin

Several mechanisms contribute to the development of resistance to platinum-based chemotherapy, including reduced drug uptake, increased drug inactivation, and decreased DNA repair, all of which reduce apoptosis in cancer cells [62]. As previously noted, AMPK may play a role in chemotherapy resistance. In the case of cisplatin (CDDP), AMPK levels significantly increase following treatment in colon cancer cells, and inhibiting AMPK has been shown to enhance CDDP’s ability to induce apoptosis [63].

CDDP resistance has also been studied in ovarian cancer. In ovarian cancer cell lines HEY, A2780, and the latter’s CDDP-resistant subline A2780CisR, adenosine (ADO) receptors A1R and A2BR were found to be expressed and functionally active. ADO demonstrated moderate cytotoxicity and induced apoptosis in a concentration-dependent manner, as evidenced by increased sub-G1 levels and cleaved PARP, thereby promoting apoptosis.

Pre-incubating adenosine (ADO) for 48 h prior to cisplatin (CDDP) treatment significantly enhanced CDDP-induced cytotoxicity and increased apoptosis synergistically. Selective antagonists of A1R and A2BR could not inhibit the ADO-induced enhancement of CDDP cytotoxicity or apoptosis; however, dipyridamole, a nucleoside transporter inhibitor, completely blocked both effects. Mechanistically, ADO increased phosphorylated AMPK levels and reduced pS6K, effects that were also prevented by dipyridamole. These in vitro results suggest that administering ADO before CDDP could be a promising strategy to enhance CDDP efficacy and potentially overcome platinum resistance in ovarian cancer [47]. In CDDP-resistant bladder cancer cells, the calcium-binding protein-39 (*CAB39*) gene is overexpressed and linked to CDDP resistance. Suppressing *CAB39* increases sensitivity to CDDP, as resistance is mediated through the CAB39-LKB1-AMPK-LC3 pathway, which promotes autophagy to maintain mitochondrial function and reduce reactive oxygen species (ROS) levels. This autophagy process can be reversed with chloroquine administration [64].

In gastric cancer, metformin reduces ATP production and activates AMPK phosphorylation at Thr172, which increases the expression of mitophagy markers such as Parkin and PINK1. This activation provides protection against cisplatin (CDDP) cytotoxicity in gastric cancer cells. Knocking down AMPKα1 prevents CDDP resistance, indicating that this pathway plays a role in the development of CDDP resistance [65].

AMPK activation by cisplatin (CDDP) is believed to contribute to CDDP’s side effects, such as kidney injury. In cultured renal tubular epithelial cells, CDDP-induced AMPK activation over time is associated with p53 phosphorylation. The selective AMPK inhibitor, Compound C, suppressed CDDP-induced AMPK activation, p53 phosphorylation, Bax induction, and caspase 3 activation both in vitro and in a mouse model of CDDP-induced kidney injury. These findings suggest that the AMPK-p53-Bax signaling pathway plays a key role in CDDP-induced apoptosis in tubular epithelial cells, providing a potential strategy to mitigate CDDP’s side effects [66].

**Figure 1:**
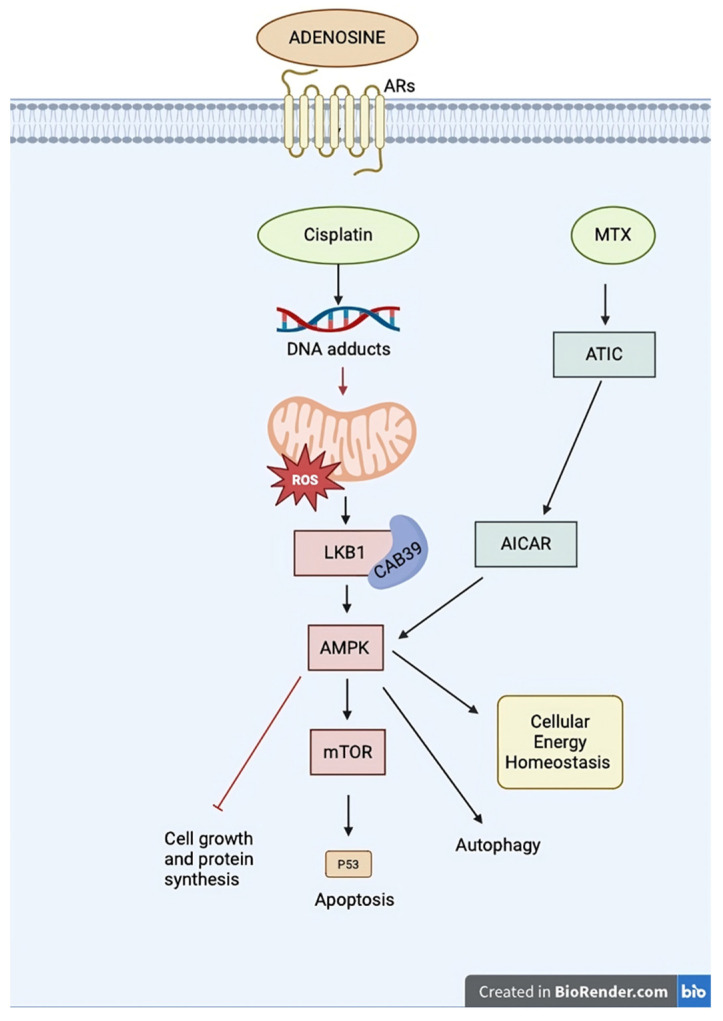
The mechanisms of resistance to cisplatin (CDDP) and methotrexate (MTX) involve multiple factors. Resistance to CDDP-based chemotherapy includes reduced drug uptake, increased inactivation, and decreased DNA repair, all of which contribute to reduced apoptosis in cancer cells. AMPK activation has also been linked to CDDP resistance in various cancer types. Additionally, combining MTX with an adenosine (ADO) pathway inhibitor is hypothesized to enhance responses to immune therapies. AICAR, aminoimidazole carboxamide ribonucleotide; AMPK, 5′-adenosine monophosphate-activated protein kinase; ARs, adenosine receptors; ATIC, 5-aminoimidazole-4-carboxamide ribonucleotide formyltransferase/IMP cyclohydrolase; CAB39, Calcium-Binding Protein 39; DNA, Deoxyribonucleic Acid; LKB1, liver kinase B1; mTOR, mammalian target of rapamycin; MTX, methotrexate; ROS, reactive oxygen species.

Although specific data on the relationship between AMPK activation and cisplatin (CDDP) resistance in sarcomas is lacking, existing evidence on AMPK activation in sarcomas (as presented in the first section of our review) and the positive outcomes of AMPK modulation to enhance CDDP response in other cancers suggest that AMPK modulation could be a promising strategy to improve treatment responses in sarcoma tumors [67]. as explained in Figure 1 of resistance to cisplatin (CDDP) and methotrexate (MTX) involve multiple factors

### 4.2. Doxorubicin

The adenosine (ADO) pathway in relation to anthracyclines has primarily been studied for its cardioprotective effects. However, some studies suggest that it may also play a role in drug resistance within this class of drugs [68].

In breast cancer research, Dr. Loi’s team analyzed gene expression data from 6000 breast cancer (BC) patients, identifying an association between CD73 expression and poor prognosis in triple-negative breast cancer (TNBC) patients. Their investigations also explored the potential link between high CD73 expression and the efficacy of anthracyclines, finding that high CD73 expression was significantly associated with a reduced rate of complete pathological response to doxorubicin (DOX)-based preoperative chemotherapy. Using murine models, the team demonstrated that CD73 expression confers resistance to DOX by suppressing the adaptive immune response via activation of the A2A receptor (A2AR). Blocking the CD73 pathway enhanced DOX’s antitumor effects, prolonging survival in murine models with metastatic disease. These findings strongly suggest a relationship between CD73 and anthracycline response in TNBC. The researchers hypothesized that anthracyclines, like DOX, exert an antitumor effect on CD8+ T cells, dependent on extracellular ATP accumulation. In murine models injected with lymphocyte-enriched tumors, an increase in CD8+ T cells and gamma interferon (IFN-γ) was observed with DOX treatment, an effect further amplified by combining DOX with a CD73 inhibitor. The team also examined CD39 and CD73 expression in response to other drugs, such as oxaliplatin, cisplatin (CDDP), and 5-fluorouracil (5-FU), and observed increased CD39 and CD73 expression with these treatments. These results support prior analyses indicating the involvement of the CD73 pathway in CDDP treatment response [69].

Dr. Rodriguez investigated how chemotherapy modifies the tumor microenvironment (TME) and its relationship with chemoresistance in breast cancer (BC). The study evaluated P2X7 and A2A receptor (A2AR) expression and function in CD8+ T cells both before and four months after BRCA patients received neoadjuvant chemotherapy. The results revealed that in non-chemoresistant (N-CHR) patients, P2X7 expression levels increased and A2AR expression decreased in CD8+ T cells compared to in chemoresistant (CHR) patients, where P2X7 expression was reduced. These alterations were particularly pronounced in triple-negative breast cancer (TNBC) and human epidermal growth factor receptor 2 (HER2)-enriched subtypes [70].

Anthracyclines are essential components in the chemotherapy treatment of soft-tissue and bone sarcomas. Previous studies have demonstrated CD73 as an active pathway in certain sarcoma cell lines, highlighting the importance of further investigation in human subjects. A deeper understanding of these pathways in humans is crucial for developing studies that could provide valuable data on the potential of CD73 inhibition to enhance treatment responses in sarcoma patients.

### 4.3. Methotrexate

Methotrexate is a well-established analog of the B vitamin folic acid. It acts as a folic acid antagonist and has been widely used as an anticancer drug. It is commonly used to treat OS, particularly in sarcomas. Additionally, it is known for its immunosuppressive and anti-inflammatory effects in rheumatoid arthritis and other autoimmune disorders [71].

Solute carrier (SLC) transporters and ATP-binding cassette (ABC) transporters are essential for methotrexate (MTX) absorption, distribution, and renal excretion. Intracellularly, the enzyme folylpolyglutamate synthase (FPGS) converts MTX into polyglutamate forms, which increases its retention and mediates pharmacodynamic effects. The level of MTX polyglutamates correlates with treatment response. MTX functions by inhibiting key enzymes involved in cell proliferation, including dihydrofolate reductase (DHFR), thymidylate synthase (TYMS), and 5-aminoimidazole-4-carboxamide ribonucleotide formyltransferase/IMP cyclohydrolase (ATIC), leading to decreased synthesis of purines, pyrimidines, and DNA. This inhibition is also associated with AMPK activation. ATIC is transported into cells via adenosine (ADO) transporters and converted by adenosine kinase into the monophosphorylated derivative AICAR, which, as noted earlier, mimics AMP in activating AMPK [72].

Beckers et al. reported that combining methotrexate (MTX) with AICAR significantly enhanced AICAR-induced AMPK activation in MCF-7 and HepG2 cells. MTX also increased the anticancer potency of AICAR, substantially raising the concentrations of AICAR and its active metabolites within tumors. The authors concluded that MTX acts synergistically with AICAR to inhibit cancer cell proliferation and tumor growth in nude mice [73].

Methotrexate (MTX) has a key mechanism of action involving the accumulation of adenosine (ADO) by inhibiting ATIC [71]. This accumulation inhibits both ADO deaminase and AMP deaminase, thereby increasing ADO concentrations, which leads to notable anti-inflammatory effects through the A2AR and A3AR receptors. Investigating the potential of MTX to enhance immunotherapy responses—either at immunomodulatory doses or in combination with ADO analogs—could be valuable in osteosarcoma (OS), as it may improve chemotherapy outcomes by activating the immune system [74]. Another promising hypothesis is that combining MTX with ADO pathway inhibitors may increase responses to immunotherapies [75]. Further research is essential to improve chemotherapy efficacy and prevent chemoresistance in sarcoma patients.

### 4.4. Gemcitabine

Gemcitabine (GEM) is commonly used to treat sarcomas, particularly when other treatments have been unsuccessful. In studies using patient-derived xenograft (PDX) models of pancreatic cancer, researchers found that the CD73 receptor is highly expressed and linked to GEM resistance, which is associated with activation of the AKT pathway. They also discovered that an AKT inhibitor could potentially reverse CD73-induced GEM resistance. In experiments with CD73 knockdown cells, adding adenosine (ADO) did not restore GEM resistance, leading the researchers to suggest that CD73’s nucleotidase activity may have a limited role in GEM resistance [76]. The researchers further explored the role of CD73 mutation in GEM resistance by creating an enzyme mutation targeting the zinc-binding site, which impaired CD73’s nucleotidase activity. However, the mutant CD73, similar to the wild-type protein, still promoted GEM resistance through AKT activation. These findings strongly suggest that CD73 may enhance GEM resistance via a mechanism independent of its nucleotidase activity [76].

The authors also examined cytosolic CD73 staining in pancreatic ductal adenocarcinoma (PDAC) patient specimens, finding substantial amounts of intracellular CD73 localized to the endoplasmic reticulum membrane. They discovered that this intracellular CD73 interacts with major vault protein (MVP) to activate the SRC-AKT pathway. Additionally, they found that troglitazone, a PGC-1α agonist, inhibits CD73 expression and enhances sensitivity to gemcitabine (GEM) by downregulating CD73 in PDAC [76]. A separate research group reported similar findings, noting that CD73 overexpression in PDAC cells promotes metastasis independently of its nucleotidase function. This group suggested that monoclonal antibodies or small-molecule inhibitors targeting CD73’s enzymatic activity may not effectively control its function. They further observed that CD73 contributes to PDAC metastasis by binding to the E3 ligase Tripartite Motif-Containing 21 (TRIM21) and competing with Snail for the binding site. In their study, adding diclofenac—a non-steroidal anti-inflammatory drug—showed greater effectiveness against PDAC metastasis than CD73-blocking antibodies and enhanced GEM’s therapeutic effects in a spontaneous KPC (LSL-KrasG12D/+, LSL-Trp53R172H/+, and Pdx-1-Cre) pancreatic cancer model [77].

### 4.5. Docetaxel

**Figure 2:**
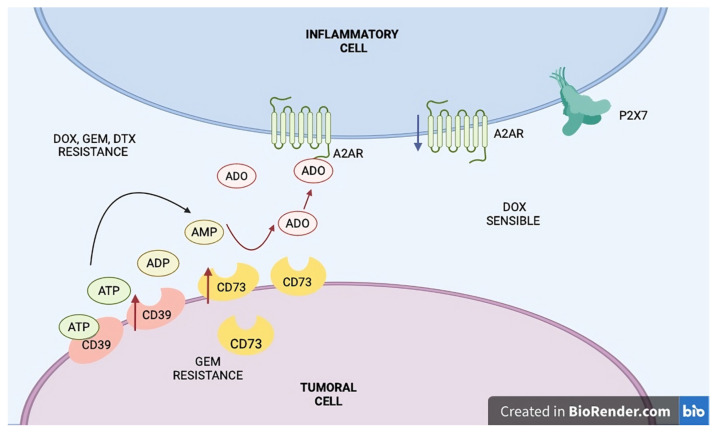
Resistance to doxorubicin, gemcitabine, and docetaxel; the mechanisms of resistance to these drugs include various cellular adaptations and pathway modulations. In the tumor cell, ATP is converted to AMP and then to ADO by the enzymes CD39 and CD73 (black arrows), which contributes to resistance to GEM. The produced ADO is transported to the inflammatory cell (red arrows), where it interacts with A2AR receptors, promoting resistance to DOX, GEM, and DTX. The A2AR-mediated DOX-sensitive state (blue arrows) suggests a potential role of ADO signaling in influencing the response to this treatment. ADO, adenosine; ADP, adenosine diphosphate; AMP, adenosine monophosphate; ATP, adenosine triphosphate; CD39, ectonucleoside triphosphate diphosphohydrolase 1; CD73, ecto-5′-nucleotidase; GEM, gemcitabine; DOX, doxorubicin; DTX, docetaxel; P2X7, purinergic receptor P2X, ligand-gated ion channel 7; A2AR, adenosine A2A receptor.

Docetaxel (DTX) is an important drug for treating sarcomas, and research has explored the potential of combining the adenosine (ADO) pathway with DTX. Studies show that DTX can increase CD73 expression and enzymatic activity in patient-derived ovarian epithelial and CD8+ T cells, leading to an immunosuppressive response that can be reversed by anti-CD73 (aCD73) antibodies. Additionally, combining DTX with aCD73 in mice significantly reduced tumor growth and lung metastasis. Another strategy involves combining DTX with the A2BR antagonist BAY606583, which inhibits the ERK signaling pathway and has shown antiproliferative effects on esophageal cancer cells [78]. Efforts to improve DTX efficacy include encapsulating it in biodegradable poly(lactic-co-glycolic acid) (PLGA) nanoparticles for treating non-small-cell lung cancer (NSCLC). ADO was conjugated to the nanoparticle surface using EDC-NHS chemistry, capitalizing on the overexpression of ADO receptors in NSCLC cells. In vitro tests of these nanoparticles assessed their physical and chemical properties, cellular uptake, and biocompatibility. DTX nanoparticles (DPLGA) exhibited changes in size and zeta potential after ADO conjugation, measuring 158.2 ± 6.3 nm and −11.7 ± 1.4 mV, respectively. Adenosine–DPLGA demonstrated higher cellular uptake in NSCLC cell lines than DPLGA, indicating that ADO receptors facilitate nanoparticle uptake [79]. This approach may also be effective in other solid tumors, such as sarcomas, to enhance drug efficacy. Similar strategies have been applied in sarcomas using other agents, such as doxorubicin delivered with pegylated nanoparticles, which have shown positive clinical outcomes and are included in clinical guidelines [80,81,82], as explained in Figure 2 of resistance to doxorubicin, gemcitabine, and docetaxel.

## 5. Adenosine and Sarcoma Microenvironment

Adenosine pathways significantly impact the tumor microenvironment. A study on tumor-derived microvesicles (MVs) from sarcoma patients showed that these particles could induce neoangiogenesis in nontumor tissues. The increase in cytosolic calcium levels facilitated new vascular structure formation by activating the P2XR4 receptor, enhancing mitochondrial activity through calcium influx and ATP consumption via the P2XR4 pathway [83].

This group conducted a differential proteomic analysis, revealing that P2XR4 is the only purinergic receptor upregulated in endothelial cells upon stimulation by tumor-derived MVs. Stimulation by sarcoma-derived MVs induces the translocation and clustering of lysosomal P2XR4 on the cell membrane, initiating a feed-forward mechanism that further elevates intracellular calcium, mitochondrial activity, and ATP production. The activity of the P2XR4 receptor appears essential for promoting cell migration and the formation of branching tubular networks [83].

Furthermore, this research group observed elevated levels of the Del-1 protein in microvesicles from tumor patients compared to healthy controls, reinforcing its proposed role in angiogenesis. Del-1, along with the proangiogenic factors CCL5 and CXCL12, polarizes P2XR4 on cell membranes, suggesting that P2XR4 may also play a role in cell motility [83].

**Figure 3:**
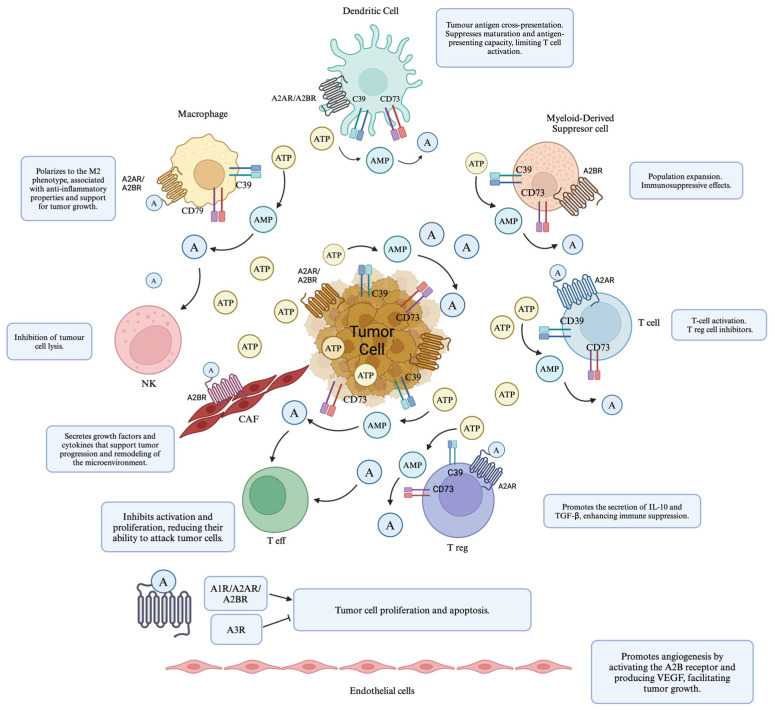
The effects of adenosine on its receptors and tumor cells in the tumor microenvironment. Adenosine, generated from ATP and AMP by the enzymes CD39 and CD73, interacts with the A2A and A2B receptors on tumor cells and cells within the tumor microenvironment. Activation of these receptors contributes to an immunosuppressive environment by inhibiting the function of effector T cells and NK cells, while enhancing the activity of regulatory T cells and M2 macrophages, promoting the secretion of immunosuppressive cytokines such as IL-10 and TGF-β. Additionally, signaling through the A2B receptor on endothelial cells stimulates angiogenesis by producing VEGF, supporting tumor growth. The figure illustrates how these interactions contribute to immune evasion, tumor growth, and modulation of the microenvironment under hypoxic and cellular stress conditions. A2AR (adenosine A2A receptor), A2BR (adenosine A2B receptor), A3R (adenosine A3 receptor), AMP (adenosine monophosphate), ATP (adenosine triphosphate), CAF (Cancer-Associated Fibroblast), CD39 (ectonucleoside triphosphate diphosphohydrolase 1), CD73 (ecto-5′-nucleotidase), IL-10 (interleukin-10), NK (natural killer cell), T eff (effector T cell), T reg (regulatory T cell), TGF-β (transforming growth factor-Beta), and VEGF (vascular endothelial growth factor).

Their study provides insights into how adenosine pathways may influence vascular formation in sarcomas. Additionally, as detailed below, this pathway is integral to the tumor microenvironment, as explained in Figure 3.

## 6. Adenosine Pathway: Emerging Targets in Sarcoma Immunotherapy

The adenosine (ADO) pathway plays a critical role in immune system regulation. Extracellular ADO can function as both an immune stimulator and suppressor, depending on the context and the signaling pathways involved [40]. In cancerous and inflamed tissues, extracellular ADO primarily exerts an immunosuppressive effect through overstimulation of Gs protein-coupled A2A receptors (A2ARs) on immune cells [42,84]. This Gs signaling, initiated by extracellular ADO, activates adenylate cyclase, leading to intracellular accumulation of cyclic AMP (cAMP). Elevated cAMP levels activate cAMP-dependent protein kinase A (PKA), which then inhibits T-cell receptor (TCR)-dependent transmembrane signaling, thereby suppressing immune cell activation. Consequently, the A2AR signaling cascade directly inhibits TCR activation, fostering an immunosuppressive environment. This signaling also increases the transcription of immunosuppressive genes in neighboring cells, as PKA phosphorylates cAMP response element-binding protein (CREB). Under hypoxic conditions, hypoxia-inducible factor-1 alpha (HIF-1α) induces the accumulation of extracellular ADO and activates the hypoxia response and cAMP response elements, promoting the production of immunosuppressive cytokines and molecules such as transforming growth factor-beta (TGF-β), interleukin-10 (IL-10), and cytotoxic T-lymphocyte antigen-4 (CTLA-4) [85].

Adenosine (ADO) reduces the activity of CD4+ and CD8+ T cells, natural killer cells, and dendritic cells, while enhancing the suppressive functions of regulatory T cells (Tregs) and myeloid-derived suppressor cells (MDSCs). In tumors, however, ADO’s effects can impair the antitumor activity of these immune cells [86]. The A2A receptor (A2AR) plays a protective role by preventing excessive immune responses that could damage host tissue. When activated, A2AR inhibits CD4+ and CD8+ T-cell functions and selectively reduces pro-inflammatory cytokine expression, which leads to the upregulation of PD-1. CTLA-4 further promotes T-cell tolerance by inhibiting interleukin-17 (IL-17) production, encouraging the development of Foxp3+ and LAG3+ regulatory T cells. Through A2ARs, ADO also inhibits dendritic cell function. Chemotherapy and radiation treatments increase extracellular ATP (eATP), and administering A2AR antagonists alongside these therapies could expand tumor-targeting T cells and prevent Treg induction. Such combinations are currently being explored in clinical trials with anti-PD1-PD-L1 therapies [87]. The biological effects of eATP are primarily mediated via P2 purinergic receptors, with P2X7R being the main mediator for ATP-dependent effects. In the tumor extracellular matrix, eATP is broken down by ectonucleotidases, mainly CD39 and CD73, which shift the environment from immune-stimulatory to immunosuppressive. CD39, found in dendritic cells, tumor-infiltrating Th17 lymphocytes, and M2 macrophages, converts ATP to ADP and AMP. CD73, present in T and B lymphocytes, stromal cells, and dendritic cells, further breaks AMP down into ADO. CD73 is also found in Tregs and is highly expressed in anergic CD4+ T cells [88,89].

In healthy individuals, CD73 helps maintain self-tolerance by acting as an immune-inhibitory checkpoint molecule, promoting the infiltration of regulatory immune cells like Tregs, MDSCs, and dendritic cells (DCs). This action fosters an immunosuppressive microenvironment that can support tumor growth [90]. CD73 has also been shown to encourage cell migration, invasion, and chemotherapy resistance in breast cancer [69].

Research on human osteosarcoma (OS) cell lines indicates that miR-16 indirectly reduces CD73 expression by inhibiting the transcription factors SMAD3 and SMAD4, which are associated with CD73 regulation [90,91]. In the hypoxic tumor microenvironment (TME), hypoxia can induce CD73 expression through HIF-1α, which regulates the epithelial–mesenchymal transition (EMT), leading to lung metastasis in triple-negative breast cancer [92]. Additionally, combining CD73 inhibition with an A2BR antagonist, chemotherapy, radiotherapy, anti-PD1/PD-L1 therapy, or anti-CTLA-4 has shown promise in improving cancer treatments [88].

The A2B receptor (A2BR) is involved in pathophysiological conditions linked to adenosine (ADO) release, such as ischemia and tumor hypoxia. Its roles include regulating vascular tone, cytokine release, and angiogenesis, as well as controlling angiogenic factors like vascular endothelial growth factor (VEGF), interleukin-8 (IL-8), and basic fibroblast growth factor [84]. Under hypoxic conditions, A2BR promotes the release of angiogenic factors, supporting tumor growth. Interestingly, A2BR expression on the surface of breast cancer cells inhibits ERK 1/2 phosphorylation, suggesting that an A2BR agonist may exhibit anticancer effects. The A3 adenosine receptor (A3AR) is highly expressed in tumor cells, where it induces cell cycle arrest, apoptosis, and tumor growth inhibition by modulating NF-κB and Wnt signaling pathways. This makes A3AR agonists promising candidates for anticancer therapies. However, A3AR also stimulates VEGF expression in an HIF-1α-dependent manner and increases angiopoietin 2, indicating that an A3AR antagonist may serve as an effective antiangiogenic therapy [88,93].

## 7. Clinical Trials of Immunotherapy and Inhibitors of Adenosine Pathway Including Sarcomas

Oleclumab is a human monoclonal antibody that selectively inhibits CD73’s catalytic activity by steric blocking and crosslinking CD73 dimers. Binding oleclumab to CD73 induces CD73 aggregation and internalization, which reduces free adenosine levels and prevents immunosuppression. Oleclumab, used alone or in combination with immunotherapy or chemotherapy, has shown tumor growth inhibition in various cancer models. Studies indicate that high CD73 expression correlates with poor prognosis in advanced colorectal, pancreatic, and non-small-cell lung cancers. In a first-in-human study, oleclumab, either alone or in combination with the anti-PD-L1 monoclonal antibody durvalumab, demonstrated reductions in free CD73 and CD73 expression on peripheral T cells and tumor cells. This combination exhibited a favorable safety profile, with response rates of 2.4% in colorectal cancer, 4.8% in pancreatic cancer, and 9.5% in non-small-cell lung cancer, and 6-month progression-free survival (PFS) rates of 5.4%, 13.2%, and 16%, respectively [94]. In a phase 2 trial for unresectable stage III non-small-cell lung cancer, adding oleclumab to durvalumab increased the objective response rate (ORR) from 17.9% to 30% and significantly extended PFS, with 12-month PFS rates rising from 33.9% to 62.6% [95,96].

In the phase 2 study (NCT04668300), patients with recurrent or metastatic angiosarcoma (cohort 1), dedifferentiated liposarcoma (DLPS) (cohort 2), and recurrent or metastatic osteosarcoma (OS) (cohort 3) were eligible if they had received at least one prior systemic therapy but had not been treated with checkpoint inhibitors. These patients also needed to have measurable disease. They received treatment with oleclumab, a selective monoclonal antibody (mAb) that inhibits CD73, and durvalumab, an mAb that blocks PD-L1. The primary efficacy endpoint for cohorts 1 and 2 was the response rate (RR) at 4 months (per RECIST 1.1), while for cohort 3, it was the event-free survival (EFS) rate at 4 months. This study included core needle biopsies and blood samples, with fresh flow cytometry to assess T-cell activation, proliferation, and function, and immunohistochemistry (IHC) to assess CD73 expression in the membrane and cytoplasm. Tumor-infiltrating lymphocytes and PD-1/PD-L1 axis engagement were analyzed through multiplex immunofluorescence staining [97]. Another trial, the phase 1/1b open-label study (NCT03454451), evaluates dose escalation and dose expansion of CPI-006, a humanized mAb targeting CD73, in adults with select advanced cancers, including sarcomas. CPI-006 is being tested as a single agent, in combination with anti-PD-L1 therapy, and with chemotherapy. This study aims to assess the safety, tolerability, pharmacokinetics, pharmacodynamics, and preliminary antitumor activity of CPI-006 in this patient population [98]. Other clinical trials are exploring adenosine inhibitors in cancer, with this review highlighting those specifically involving sarcoma patients, as summarized in Table 1.

## 8. Nanoparticles as Easier Delivery of Adenosine Inhibitors

Despite efforts to develop drugs that modulate the adenosine pathway, challenges such as limited half-life and low bioavailability have hindered their clinical application. Using nanoparticles as delivery vehicles can enhance the efficacy of these inhibitors by extending their circulation time and improving their target specificity, thereby reducing the toxic effects [99]. Common types of nanoparticles used include liposomes, nanoproducts, inorganic carriers, hydrogels, biomimetic carriers, and cationic polymer nanocarriers. As discussed, nanoparticles like pegylated liposomal doxorubicin and liposomal mifamurtide have shown effectiveness in treating sarcomas. Currently, three-dimensional systems are being developed to further enhance treatment delivery and improve penetration across the barriers of the tumor microenvironment and extracellular matrix [82].

Additionally, for small-molecule-based drugs, nanocarriers protect these compounds, allowing for controlled and targeted release that enhances therapeutic response. Systems like pegylated liposomal doxorubicin are effective in sarcoma treatments and can be adapted for delivering adenosine pathway inhibitors. Protein- and gene-based drugs (such as siRNAs and cDNAs) are highly sensitive to environmental factors and therefore require specialized carriers, such as polymeric nanoparticles or virus-like particles. Encapsulation within nanoparticles not only stabilizes these proteins but also improves their solubility and reduces adverse effects. Injectable hydrogels that combine proteins like ADA with chemotherapy agents, such as doxorubicin, have shown promise by converting adenosine to inosine, leading to significant antitumor effects [100,101].

Furthermore, combining adenosine pathway inhibitors with therapies such as chemotherapy, radiation, photothermal therapy, and photodynamic therapy has shown substantial clinical benefits. Nanoparticle delivery systems enhance the specificity of chemotherapy, reducing toxicity and adverse effects on healthy cells. For example, mesoporous silica nanoparticles coated with macrophage membranes and loaded with catalytic agents and doxorubicin optimize immune responses by blocking A2AR and promoting dendritic cell maturation, which boosts CD8+ T-cell activity. In combination therapy, CD73 inhibition has been paired with immune checkpoint inhibitors, such as PD-1 and PD-L1 blockers, to improve immunotherapy efficacy in preclinical trials. These advancements underscore the critical role of nanoparticles as delivery vehicles, enabling the simultaneous administration of multiple drugs and immunomodulatory agents and paving the way for more effective cancer immunotherapy [99,101].

## 9. Conclusions

Sarcomas are highly diverse tumors, leading to various mechanisms of drug resistance. They often exhibit limited response to treatments and can rapidly develop resistance to therapies. Commonly classified as “cold tumors”, sarcomas generally respond poorly to current immunotherapy strategies. This review highlights the role of adenosine pathways in the resistance mechanisms associated with the primary drugs used to treat sarcomas. These pathways also contribute to the immunosuppressive environment observed in many sarcoma subtypes. Thus, developing therapeutic strategies to modulate adenosine pathways is essential for enhancing treatment responses and offering more options for patients with this challenging disease. Targeting adenosine represents a promising strategy to overcome resistance to systemic therapies in sarcoma patients. Additionally, modulating the adenosine pathway is being investigated as a method to enhance immunotherapy responses in sarcomas. Further research is needed to clarify the potential role of adenosine signaling and its inhibitors in mitigating chemotherapy resistance in these tumors.

## Figures and Tables

**Table 1:** Adenosine therapy in sarcoma.

Study	Population	Intervention	Objectives	Trial Number	Reference
Phase II study	- Patients 12 years and older with metastatic sarcoma showing disease progression or refractory disease. - Types: angiosarcoma, dedifferentiated liposarcoma, osteosarcoma	- Durvalumab (anti-PD-L1 monoclonal antibody) - Oleclumab (anti-CD73 monoclonal antibody)	Primary: - Efficacy - Tumor response - EFS Secondary: - PFS - OS	NCT04668300	[84]
Phase 1/1b multicenter study	- Patients 18 years and older with diverse advanced cancer types, including sarcomas	- CPI-006 (anti-CD73 monoclonal antibody) - CPI-006 + ciforadenant (oral A2A antagonist) - CPI-006 + pembrolizumab	Primary: - Dose-limiting toxicities - Incidence of adverse events Secondary: - Maximum dose level - OR	NCT0345445	[85]

CPI-006 (anti-CD73 monoclonal antibody); ciforadenant (oral adenosine A2A antagonist); EFS, event-free survival; OR, objective response; OS, overall survival; PFS, progression-free survival.

## Data Availability

Not applicable.
